# Nanoparticle-based delivery systems modulate the tumor microenvironment in pancreatic cancer for enhanced therapy

**DOI:** 10.1186/s12951-021-01134-6

**Published:** 2021-11-22

**Authors:** Ming Jia, Dan Zhang, Chunxiang Zhang, Chunhong Li

**Affiliations:** 1 Department of Pharmaceutical Sciences, School of Pharmacy, Southwest Medical University, No.1, Section 1, Xianglin Road, Luzhou, Sichuan 646000 People’s Republic of China; 2 Department of Pharmacy of Traditional Chinese Medicine, School of Pharmacy, Southwest Medical University, Luzhou, 646000 Sichuan China; 3 The Key Laboratory of Medical Electrophysiology of the Ministry of Education, Southwest Medical University, No.1, Section 1, Xianglin Road, Luzhou, Sichuan 646000 People’s Republic of China

**Keywords:** Pancreatic cancer, Tumor microenvironment, Nano-delivery systems, Cancer-associated fibroblasts, Extracellular matrix, Immunosuppression

## Abstract

Pancreatic cancer is one of the most lethal malignant tumors with a low survival rate, partly because the tumor microenvironment (TME), which consists of extracellular matrix (ECM), cancer-associated fibroblasts (CAFs), immune cells, and vascular systems, prevents effective drug delivery and chemoradiotherapy. Thus, modulating the microenvironment of pancreatic cancer is considered a promising therapeutic approach. Since nanoparticles are one of the most effective cancer treatment strategies, several nano-delivery platforms have been developed to regulate the TME and enhance treatment. Here, we summarize the latest advances in nano-delivery systems that alter the TME in pancreatic cancer by depleting ECM, inhibiting CAFs, reversing immunosuppression, promoting angiogenesis, or improving the hypoxic environment. We also discuss promising new targets for such systems. This review is expected to improve our understanding of how to modulate the pancreatic cancer microenvironment and guide the development of new therapies.

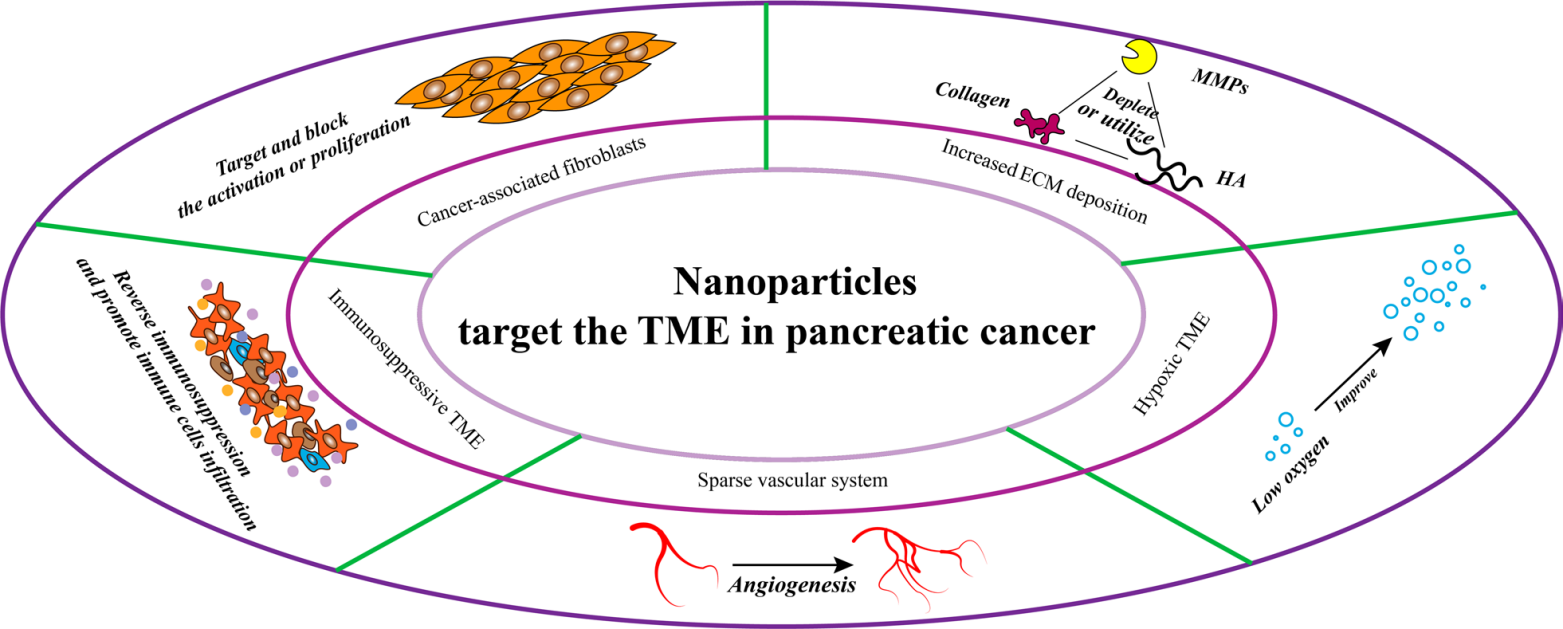

## Introduction

**Fig. 1:**
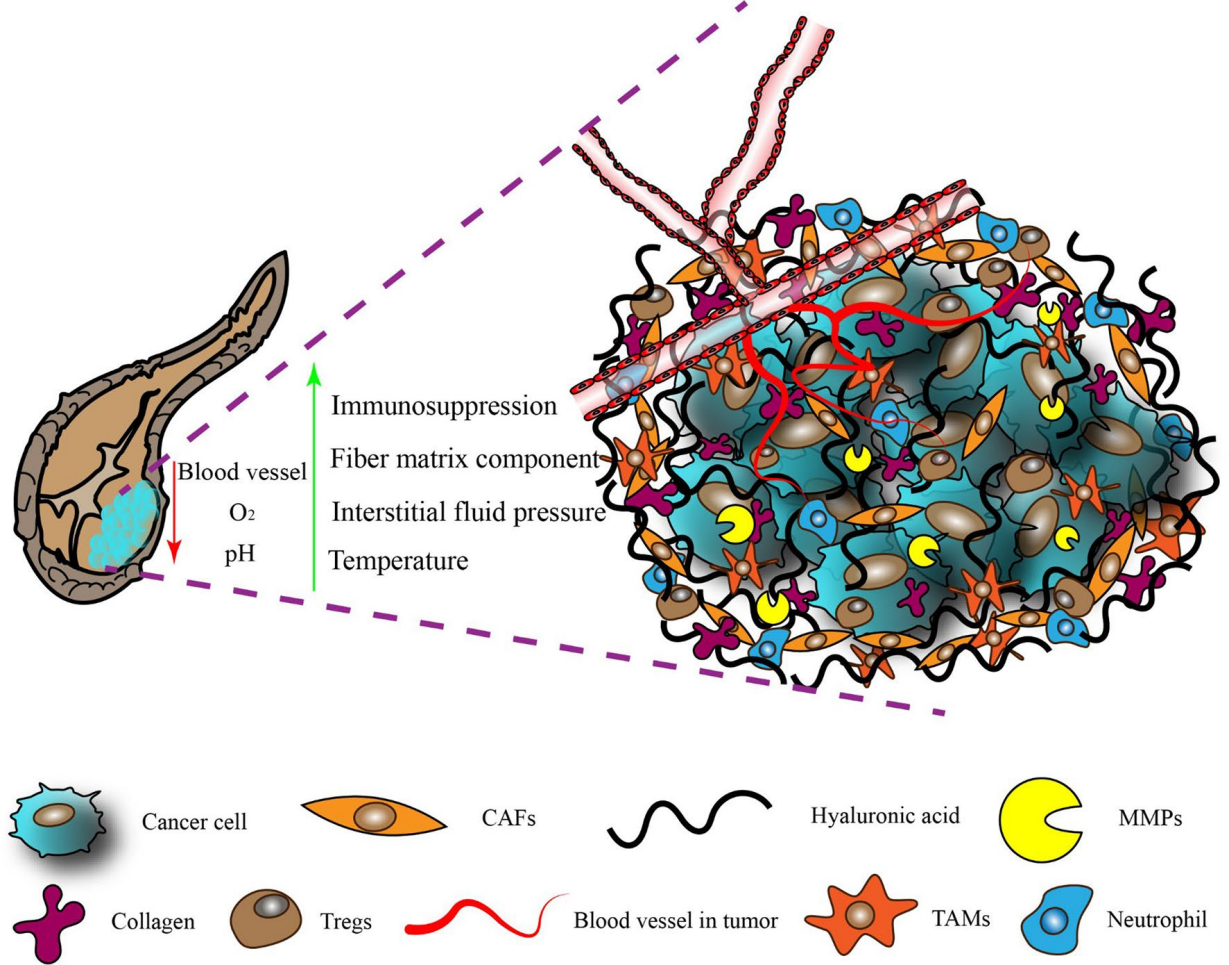
The tumor microenvironment (TME) in pancreatic cancer. The expanded extracellular matrix increases interstitial stress, while collapse of blood vessels reduces oxygen levels and acidifies the pH. Low infiltration by immune cells and their phenotypic transformation inhibit anti-tumor immune responses. Various activation pathways generate cancer-associated fibroblasts (CAFs), key cellular components of the TME. MMPs, matrix metalloproteinases; TAMs, tumor-associated macrophages; Tregs, regulatory T cells

Pancreatic cancer is one of the most aggressive and fatal malignant tumors, with a five-year survival rate of less than 5%. Fully 90% of cases occur as pancreatic ductal adenocarcinoma (PDAC). This cancer type progresses rapidly, it responds weakly to treatment, and patient prognosis is poor. In addition, metastasis reduces survival to less than half a year [1–4]. There are no biomarkers specific for pancreatic cancer, and biomarkers for other cancers do not recognize it reliably [5, 6]. Pancreatic cancer cells can adapt gradually to the nutrient-deficient environment induced by the desmoplastic reaction and develop stem cell-like properties to meet their energy needs [7–9]. Excessive stroma caused by desmoplastic reaction is the main feature of pancreatic cancer TME that is rarely observed in other malignant tumors [10]. Dense fibrous stroma tightly surrounds the cancer cells [11, 12], one of the defining histopathological features of pancreatic cancer, which constitutes more than 80% of the tumor mass [13] and is much higher than that of other tumors [14]. On the one hand, the fibrous stroma is so dense that the vasculature becomes extremely sparse, resulting in anti-angiogenic therapy that is suitable for most tumors but not for pancreatic cancer [15]. On the other hand, unlike other tumors, the tumor stroma of pancreatic cancer seems to act as a natural barrier between the body's immune system and the tumor, limiting the use of immunotherapy [16, 17]. Most importantly, the barrier protects tumor cells from being attacked by conventional chemotherapy drugs to develop chemoresistance [18]. Therefore, when designing therapeutic strategies for pancreatic cancer, we have to consider the unique TME (Fig. 1).

Various nano-delivery systems have been developed and widely used for diagnosing and treating various cancers [19, 20]. Nano-delivery systems can increase biocompatibility and solubility and prolong time in circulation [21], while their facile modification and multifunctional properties enable further optimization, such as for targeting particular cells. The ischemic and hypoxic TME consists of ECM components, as well as cells and cytokines closely related to growth, invasion, and metastasis [22, 23]. Nano-delivery systems have been developed to act on these components or stimulate certain changes in order to inhibit the survival and growth of pancreatic cancer cells.

## Extracellular matrix (ECM)

**Fig. 2:**
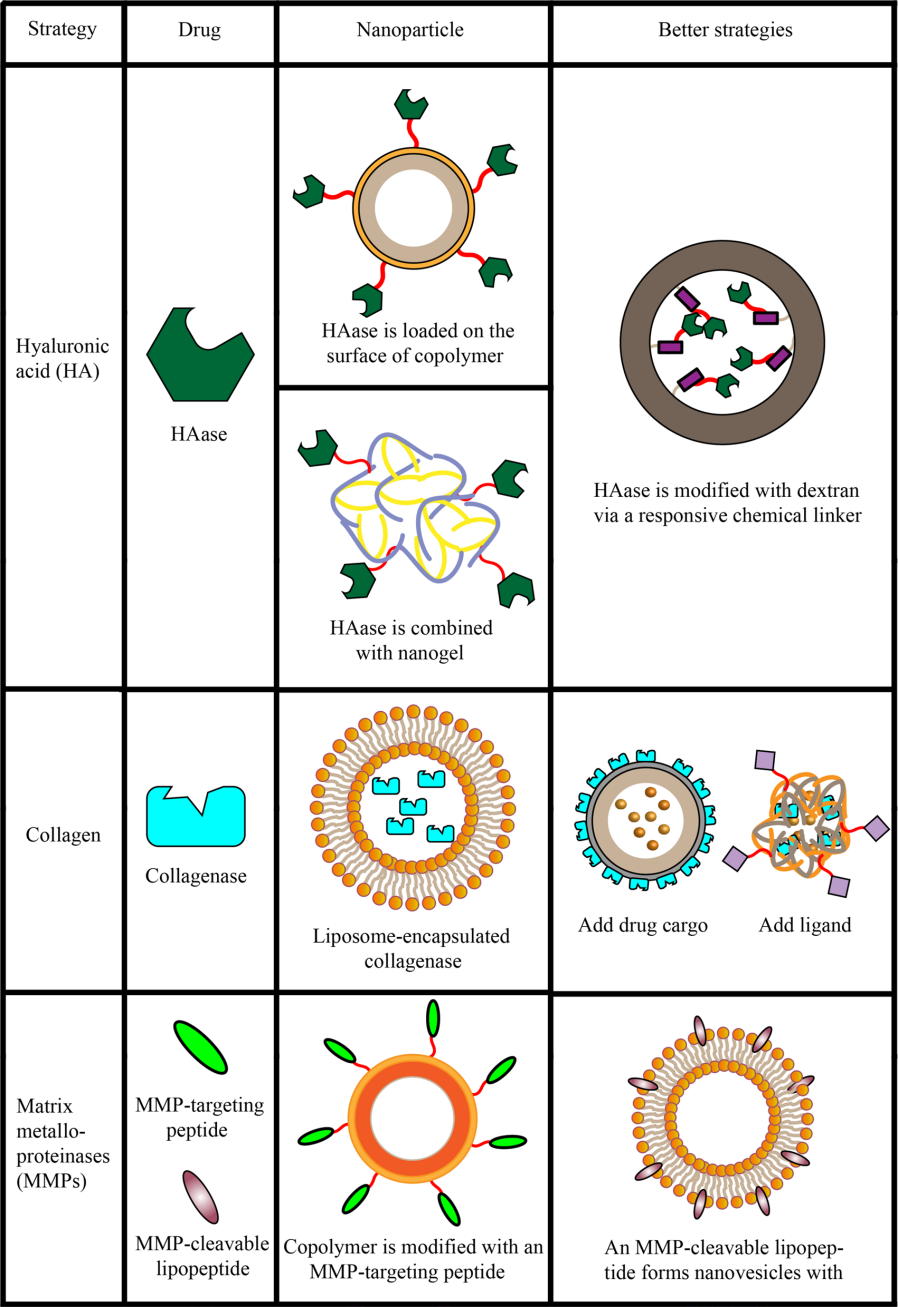
Nanoparticle delivery systems used to target the extracellular matrix in the tumor microenvironment

The ECM is a dense network that consists of collagen, fibronectin, proteoglycan, hyaluronic acid (HA), catalytic enzymes, and proteases. It is is present in all tissues, where it helps maintain structure and biochemistry [24, 25]. All ECM components interact closely with various cells, forming a dynamic microsite with various functional states [26]. In contrast to other tumors [27–29], the increased ECM deposition in pancreatic cancer inhibits immune cell infiltration and drug delivery to the tumor core [30]. For example, only 0.7% of the administered nanoparticle dose can be delivered to the solid tumor site [31]. Abnormally overexpressed matrix components may lead to strong invasive metastasis, while maintaining a specific environment that promotes cancer cell proliferation [32, 33]. This connective tissue hyperplasia is very important for the pathogenesis of pancreatic cancer, indicating that ECM modulation may be a promising treatment strategy for this type of cancer (Fig. 2).

### Hyaluronic acid (HA)

HA, a key component of the ECM, is a hydrophilic glucosamine polysaccharide that is strongly expressed in pancreatic tumors. It reduces tumor perfusion and infiltration by anti-tumor immune cells, while increasing tumor interstitial fluid pressure and vascular collapse [34]. HA has been closely associated with tumor proliferation and metastasis [35]. Median overall survival of pancreatic cancer patients is 15 months shorter among those showing high HA deposition than among those with low deposition [36]. This suggests that HA accumulation is one of the main factors for poor prognosis in patients with pancreatic cancer.

Hyaluronidase (HAase) is an endogenous degradation enzyme that quickly transforms HA into oligosaccharides and low-molecular-weight HA. Studies in genetically engineered mouse models of PDAC showed that combining gemcitabine with PEGylated recombinant human HAase PH20 (PEGPH20), which can rapidly and sustainably deplete HA, can increase tumor perfusion and vascular permeability, even more so than gemcitabine alone [37]. In fact, this combination therapy has proven effective even against stage IV pancreatic cancer involving high HA deposition [9]. These results indicate that HA depletion can promote drug delivery to the tumor site. However, HAase can be inactivated and degraded in vivo*,* reducing its half-life in serum and limiting its accumulation at the tumor site.

Therefore, nanoparticles (NPs) have been used to protect the enzyme from side effects and improve its tumor delivery. For instance, recombinant human HAase PH20 (rHuPH20) was loaded on the surface of poly(lactic-*co*-glycolic acid)-*b*-polyethylene glycol (PLGA–PEG) NPs, which were then coated with an additional PEG layer to protect the rHuPH20 and improve its accumulation at the tumor site [38]. In another study, HAase was chemically modified to produce thiols, and this enzyme was combined with nanogels for protection and improved delivery at the tumor site [39]. However, those previous studies conjugated linear PEG chains to HAase via non-cleavable bonds, which can affect enzyme activity. Thus, HAase was modified with dextran via a chemical linker, which could dissociate within the acidic TME, restoring the enzyme’s activity [40]. Clinical studies confirmed the ability of HAase to promote drug absorption and penetration in tumor tissues expressing high HA [26, 41].

### Collagen

Collagen is another major component of ECM that is involved in the fibrosis of the pancreatic cancer microenvironment. Excessive production of collagen can lead to drug resistance and limit drug absorption [42]. Analogously to HA depletion from ECM, collagen catabolism has been explored as a way to increase drug delivery [43, 44], and collagenase has been approved by the U.S. Food and Drug Administration and the European Medicines Agency for the treatment of palm fibromatosis [45]. For instance, the efficacy of chemotherapy in PDAC mice was significantly increased by pretreatment with liposome-encapsulated collagenase, indicating that collagenase can enhance tumor penetration and chemotherapy [46]. However, long intervals between chemotherapeutic drug administrations may prevent its timely delivery to the tumor site, leading to ECM destruction that may further promote cancer metastasis [47]. To avoid this risk and improve drug efficacy, collagenase and doxorubicin (DOX) were co-loaded onto PLGA NPs carrying an adhesive polydopamine layer [48]. In another approach, the monoclonal anti-HER2 antibody trastuzumab and collagenase have been formulated into a thermosensitive PLGA–PEG–PLGA hydrogel to improve drug release and treatment efficacy [49]. Nevertheless, collagenase has been applied in cancer treatment much less often than HAase, probably because its cleavage products cannot be effectively separated from collagen fibers, leading only to small local changes in the collagen structure [50].

### Matrix metalloproteinases (MMPs)

ECM also contains an abundance of proteolytic enzymes that help maintain its structure. Among them, matrix metalloproteinases (MMPs) are known to play a key role in cancer occurrence, tumor growth, and metastasis. Studies have shown that MMP-1, MMP-2, MMP-7, MMP-9, membrane type 1-MMP (MT1-MMP), MT2-MMP, and MT3-MMP are overexpressed in pancreatic cancer tumors, while MMP-2, MMP-7, and MMP-9 have been identified as potential biomarkers of pancreatic cancer [51]. NPs modified with a MT1-MMP-binding peptide showed excellent targeting and uptake ability in a pancreatic cancer mouse model [52]. In another study, an MMP-9-cleavable lipopeptide was synthesized to form nanovesicles with various lipids [53]. In the presence of elevated glutathione levels, the outer PEG groups were reductively removed, exposing the substrate lipopeptides to MMP-9. As a result, the lipid bilayer of the vesicle was disrupted, releasing the encapsulated content. In this way, MMP-9-responsive nanovesicles were able to control tumor growth more effectively than MMP-9-free vesicles, suggesting that MMPs can be used as an inducible “trigger” to enhance drug accumulation in pancreatic tumors.

## Cancer-associated fibroblasts (CAFs)

**Fig. 3:**
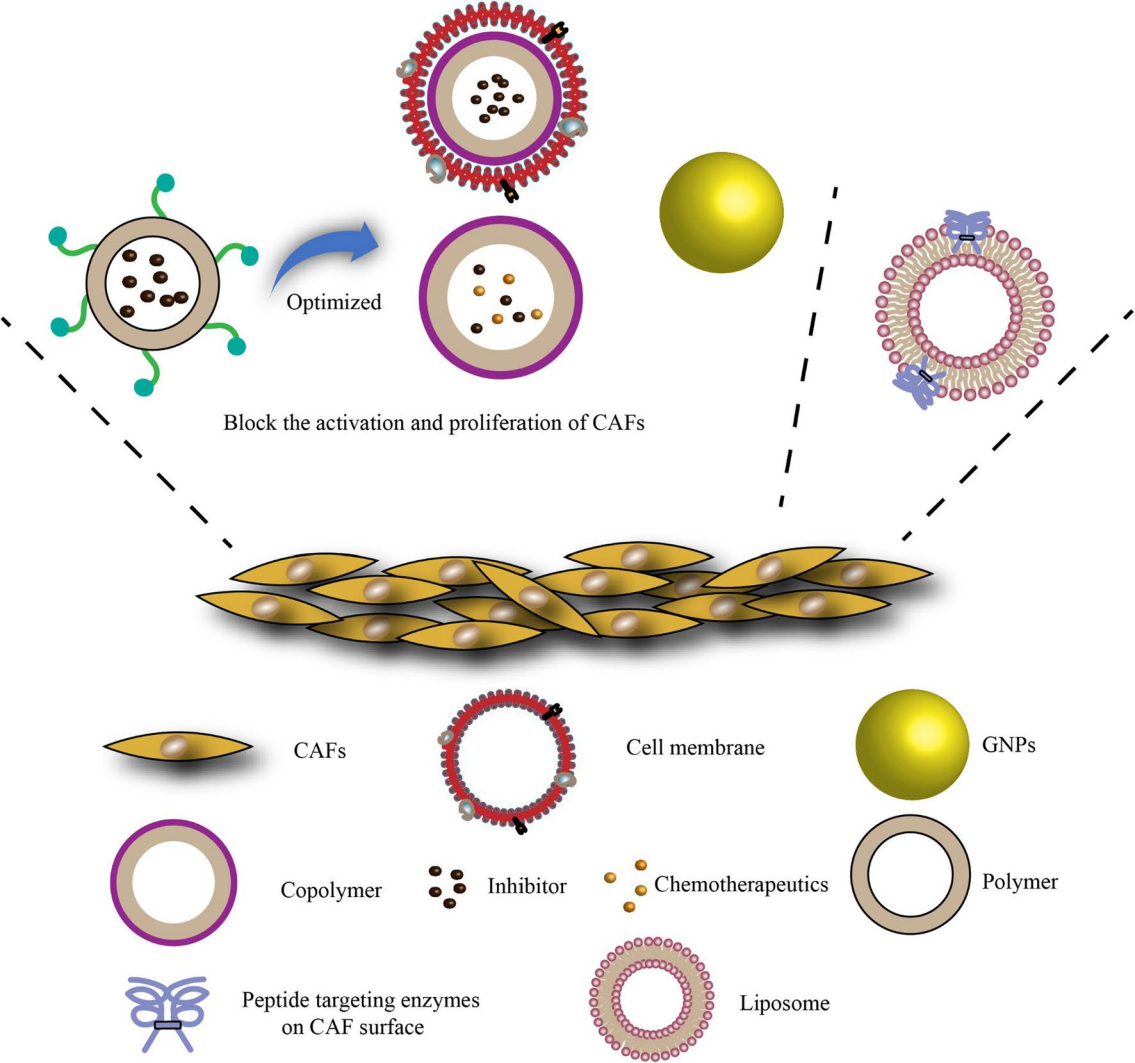
Different nanoparticle optimization strategies used to block the activation and proliferation of cancer-associated fibroblasts (CAFs). GNPs, gold nanoparticles

In normal pancreatic tissue, fibroblasts and pancreatic stellate cells are responsible for maintaining the normal structure of the glandular connective tissue [54]. However, in cancer tissues, various activation pathways generate CAFs from bone marrow mesenchymal stem cells, pancreatic stellate cells, and resting fibroblasts. These activation pathways include sonic hedgehog (SHH), transforming growth factor-β (TGF-β), tumor necrosis factor-α (TNF-α), interleukin (IL)-1, IL-6, and IL-10. CAFs are a key component of the tumor stroma, where they secrete molecules and contract [55–58]. They are densely arranged around all pancreatic tumor sites, while also appearing in some benign tissues and ducts [59–61]. Therefore, blocking the activation and proliferation of CAFs or targeting them for drug delivery may serve as a new therapeutic strategy for pancreatic cancer (Fig. 3).

Recently, CAF-targeting biodegradable polymer NPs loaded with α-mangostin and coated with CREKA peptide were developed [62]. α-Mangostin is known to modulate the TME by interfering with the TGF-β/Smad signaling pathway and blocking the activation of CAFs, while the CREKA peptide has specific affinity for fibronectin, which is overexpressed on the CAF membrane. The peptide coating enhanced the uptake of CAFs, while the combination with α-mangostin strengthened CAF inhibition, reshaping the TME in a way that compromised the stroma barrier. Similarly, the SHH inhibitor cyclopamine has been encapsulated into PLGA NPs, which were coated with erythrocyte membrane for better biocompatibility and longer circulation time [63].

However, blocking only the activation of CAFs may not effectively kill pancreatic tumors. Therefore, cyclopamine, which can deplete stroma-producing CAFs, has been encapsulated into polymer micelles together with paclitaxel (PTX), an effective inhibitor of tumor proliferation [64]. Compared to the effects of using cyclopamine alone, co-delivery of cyclopamine and a chemotherapeutic drug reduced the adverse effects of stroma ablation and led to greater therapeutic effect, which could be optimized by regulating the ratio of the two agents.

Enzymes overexpressed on the CAF membrane have also been targeted in order to optimize drug delivery. For instance, the albumin nanoparticle of PTX (HSA–PTX) was encapsulated into cleavable amphiphilic peptide (CAP)-modified liposomes that were responsive to fibroblast-activated protein-α (FAP-α), a membrane-bound serine protease specifically expressed on the surface of CAFs. CAP enhanced drug accumulation at the tumor site and promoted the enzymatic reaction with FAP-α, thus facilitating the release of HSA–PTX [56].

Interestingly, the materials used to prepare nanocarriers themselves can inhibit CAF activation. For example, gold NPs (GNPs) have been used to shift CAF phenotype from activated to stationary by inducing endogenous lipid synthesis [65], suggesting that GNPs may enable the development of functional drug nanodelivery strategies.

## Immunosuppressive TME

During the transformation of normal pancreatic tissues into cancer tissues, connective tissue hyperplasia leads to tissue fibrosis, while the numbers of tumor-associated macrophages (TAMs) of the M2 phenotype, neutrophils of the N2 phenotype, and regulatory T cells (Tregs) increase, changing the immunophenotype of the disease [54]. TAMs are one of the most abundant immune cells in the TME [66], and the ratio of TAMs with the M2 phenotype to the number with the M1 phenotype is inversely related to disease progression and survival time [67]. In the presence of T-helper 2 cell-type cytokines, M1 TAMs are polarized toward the M2 phenotype, which is immunosuppressive and promotes tumor growth [68]. In addition, the interaction of M2 TAMs with cancer cells and various non-cancer cells promotes cancer cell proliferation, drug resistance, and distant metastasis, attracting macrophages that form malignant feedback loops and continuously strengthen immunosuppression [69].

**Fig. 4:**
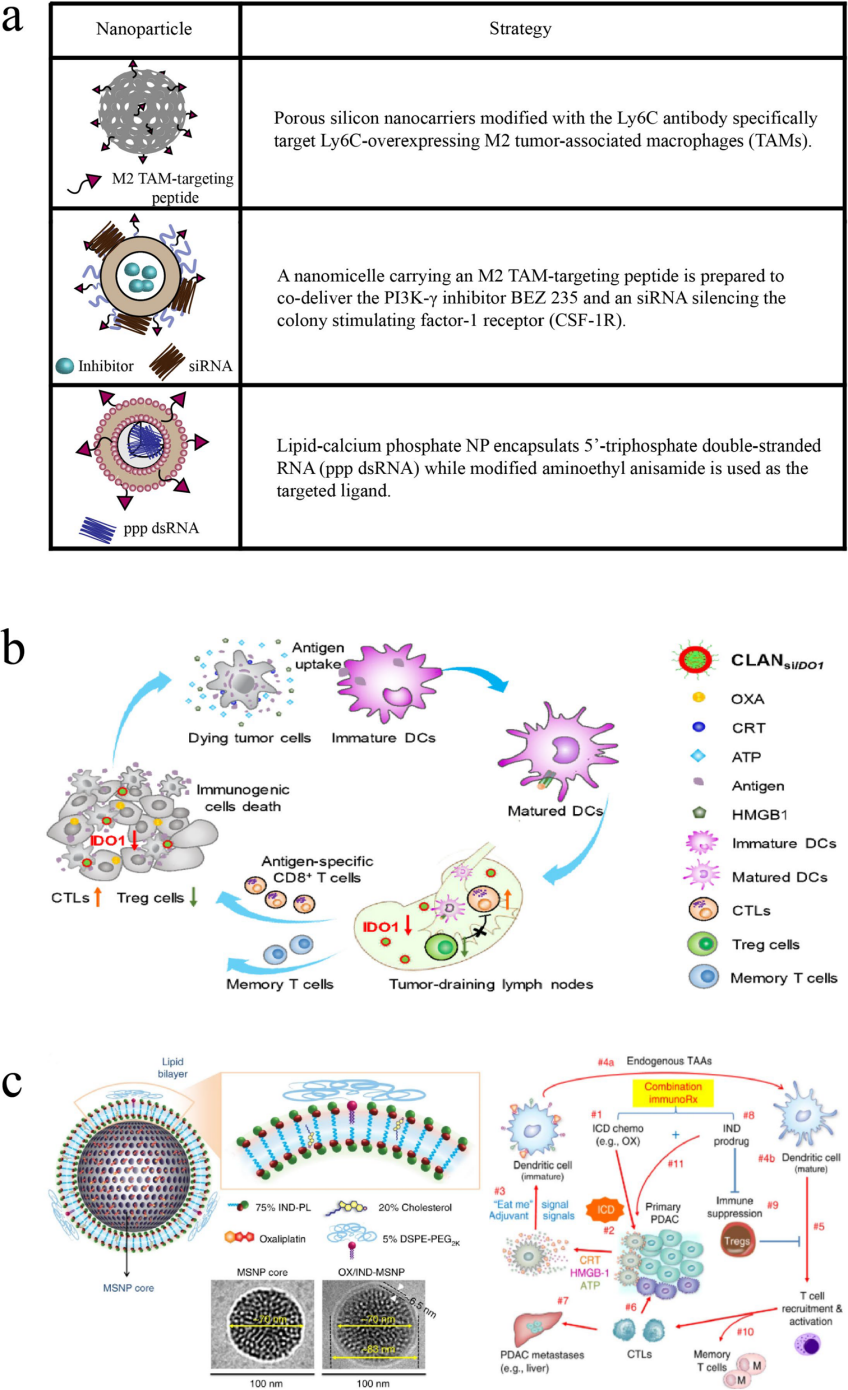
Nanoparticle optimization strategies used to reverse immunosuppression in the tumor microenvironment. **a** Some nanoparticles are used to target M2 TAMs. **b** Indoleamine 2,3-dioxygenase-1 (IDO1) siRNA-loaded lipid nanoparticles enhance immunogenic cell death (ICD) to promote immunogenic death of tumor cells. Reprinted by permission from [73]. **c** Co-encapsulation of the ICD inducer oxaliplatin and the IDO inhibitor indomethacin in mesoporous silica nanoparticles (MSNPs) coated with lipid bilayers. Reprinted by permission from [74]. *DCs* dendritic cells, *CTLs* cytotoxic T lymphocytes, *OXA* oxaliplatin, *CRT* calreticulin, *HMGB-1* high-mobility group box 1, *IND* indoximod

Ly6C, a mouse homolog of CD59, is highly expressed in TAMs. To specifically target Ly6C-overexpressing TAMs, the surface of porous silicon nanocarriers was modified with an anti-Ly6C antibody (Fig. 4a) [70]. Another study showed that the polarization of M2 TAMs is mediated by multiple signaling pathways [69], especially PI3K-γ and CSF-1/CSF-1R. Therefore, a nanomicelle carrying an M2 TAM-targeting peptide was prepared to co-deliver the PI3K-γ inhibitor BEZ 235 and an siRNA silencing the colony stimulating factor-1 receptor (CSF-1R) in order to inhibit TAMs specifically [71]. The nanomicelle inhibited both the PI3k-γ and CSF-1R pathways, while the level of M2 TAMs decreased and that of M1 TAMs increased, resulting in a remodelling of the immune microenvironment. In another therapeutic approach, NPs encapsulating 5′-triphosphate double-stranded RNA (ppp dsRNA) were developed. Lipid-calcium phosphate NPs were selected as the drug delivery vector, since they have been widely used to deliver phosphorylated biomolecules, while modified aminoethyl anisamide was used as the targeted ligand. The delivered ppp dsRNA induced cancer cell apoptosis by binding to retinoic acid-inducible gene I-like receptors to produce type I interferon and silence Bcl2, thus increasing the proportion of M1 TAMs and reducing immunosuppression in the TME [72].

Interfering with the indoleamine 2,3-dioxygenase (IDO) pathway to reverse the immunosuppressive TME has also been shown to enhance immunogenic cell death (ICD). Specifically, IDO1 siRNA encapsulated in lipid NPs reached the tumor site without excessive loss, where it downregulated Tregs to promote ICD (Fig. 4b) [73]. In a subsequent study, a more effective co-delivery nanosystem was prepared, where the ICD inducer oxaliplatin and the IDO inhibitor indomethacin were co-encapsulated in mesoporous silica NPs coated with lipid bilayers, achieving greater synergy than the previous method (Fig. 4c) [74]. These results illustrate how incorporating anti-immunosuppression strategies into NP-based drug delivery can improve therapeutic efficacy.

## Angiogenesis

Anti-angiogenesis therapies are commonly used in various cancers, including glioblastoma, non-small cell lung cancer, renal cell carcinoma, hepatocellular carcinoma, and multiple myeloma [15, 24]. However, no clinically effective anti-angiogenesis agents have been reported for pancreatic cancer, as its vascular system is affected by high interstitial fluid pressure and the microvessel density is inversely related to the interstitial surface area [24].

Cilengitide, a small molecule with strong anti-angiogenesis effects, induces endothelial cell apoptosis at micromolar concentrations, but it promotes endothelial cell migration and enhances tumor angiogenesis at nanomolar concentrations [75]. To improve its tumor-targeting ability, cilengitide-loaded nanocarriers were prepared. High cilengitide loading led to high affinity and internalization, inhibiting angiogenesis and improving the ability of chemotherapy drugs to kill endothelial cells. Low cilengitide loading prevented integrin-mediated endocytosis, but did not promote endothelial cell migration due to insufficient interaction of the drug with integrins [76–78].

**Fig. 5:**
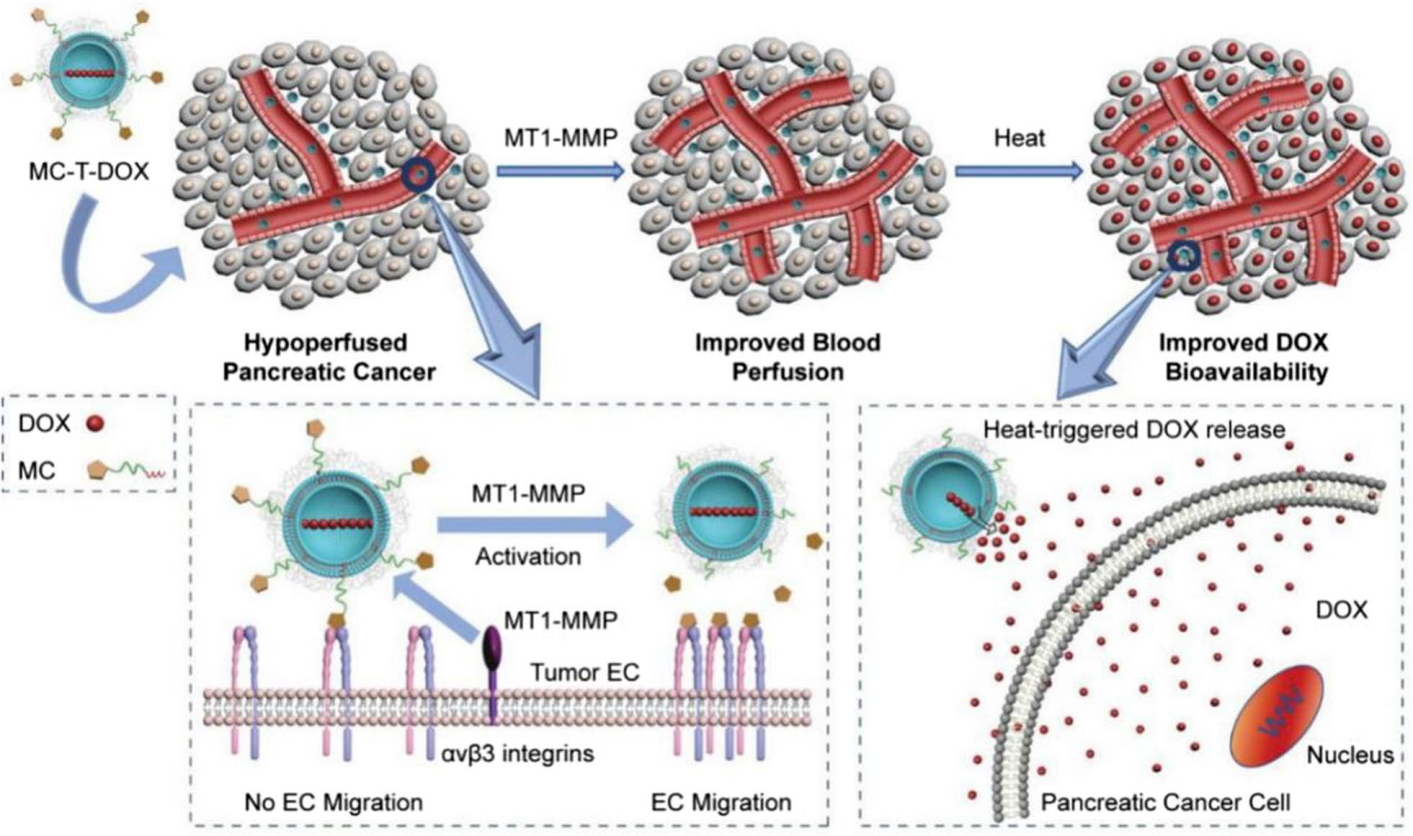
Cilengitide encapsulated in a doxorubicin (DOX)-loaded thermosensitive liposome via a MT1-MMP-cleavable peptide improves tumor blood perfusion and drug delivery in pancreatic cancer. Cilengitide is released through MT1-MMP cleavage on tumor endothelial cells (ECs), promoting EC migration and angiogenesis. MT1-MMP, membrane type 1-matrix metalloproteinase; MC, MT1-MMP-activated cilengitide. Reprinted by permission from [79]

Cilengitide was encapsulated in doxorubicin (DOX)-loaded thermosensitive liposomes via an MT1-MMP-cleavable peptide (MC-T-DOX). After reaching the tumor site, cilengitide was released through MT1-MMP cleavage on tumor endothelial cells (Fig. 5). This approach prevented excessive endothelial cell endocytosis and ensured sufficient interaction of cilengitide with α_v_β_3_ integrins on endothelial cells to form new blood vessels into ischemic areas, thereby improving the delivery of drug-loaded liposomes [79]. Compared with the previously mentioned ECM depletion strategy [37, 38, 46], which cannot be strictly controlled, this treatment promoted angiogenesis and blood perfusion, but it did not increase the risk of tumor metastasis [80, 81], suggesting that this strategy can safely address the challenge of pancreatic hypoperfusion for drug delivery.

## Hypoxia

**Fig. 6:**
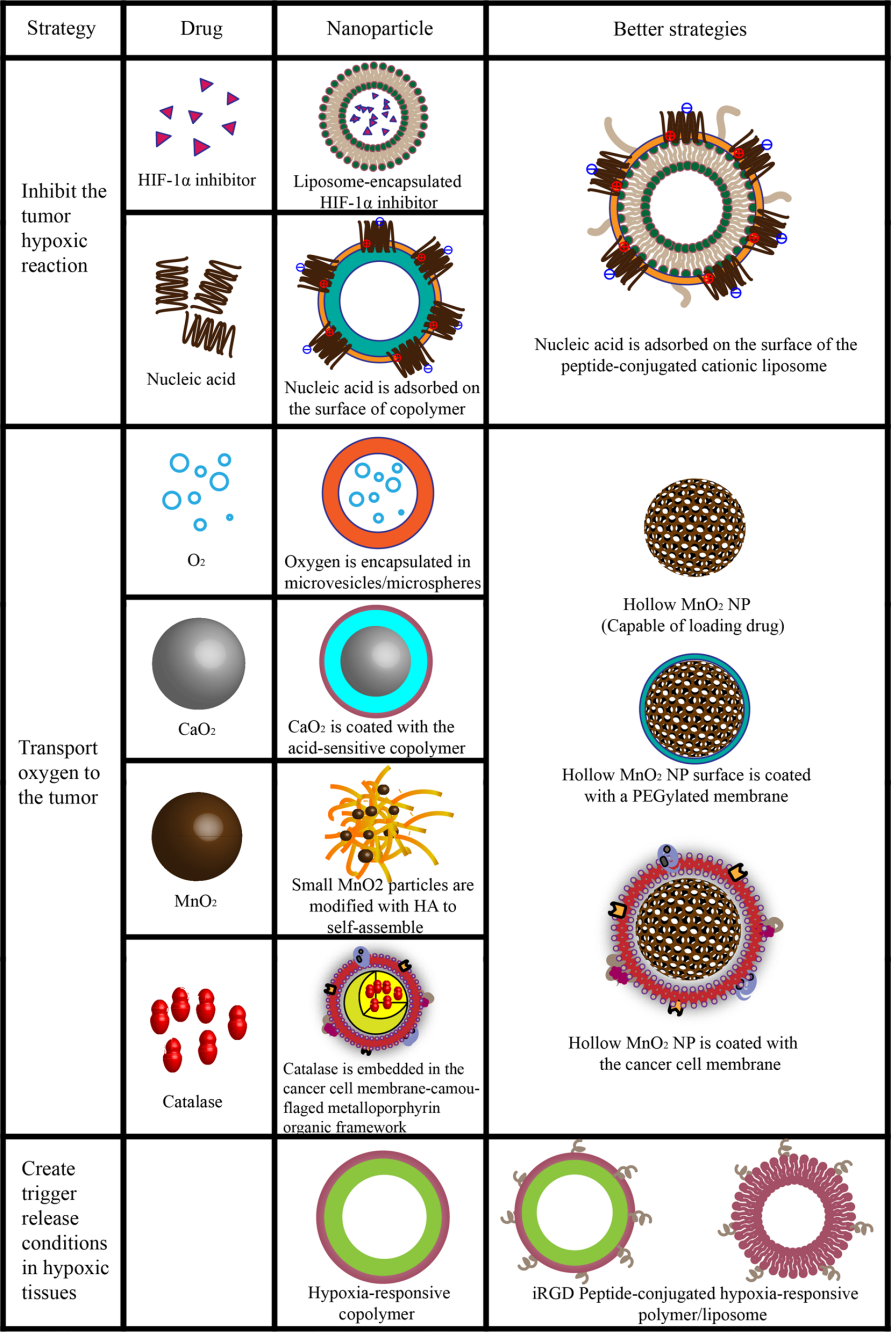
Nanoparticle-based strategies used to target hypoxia in pancreatic cancer

The highly dense ECM and sparse vascular system in pancreatic cancer limit the delivery of oxygen to the tumor site. Pancreatic cancer cells adapt to the low oxygen levels, becoming invasive and drug-resistant and metastasizing even at early stages of tumor development [82–85]. Therefore, current research targeting hypoxia in pancreatic cancer focuses on (1) inhibiting the tumor hypoxic reaction, (2) transporting oxygen to the tumor, or (3) creating trigger release conditions in hypoxic tissues (Fig. 6).

### Inhibit the tumor hypoxic reaction

Hypoxia-inducible factor-1α (HIF-1α) is considered the most promising target in the hypoxia signaling pathway to control cell metabolism, migration, and apoptosis, as well as regulate the transcription of angiogenesis growth factors [86, 87]. Therefore, the HIF-1α inhibitor YC-1 was loaded into transferrin receptor-1-targeting liposomes for delivery to pancreatic tumors to improve hypoxia and anti-tumor effects [88]. However, selective gene silencing has proven more effective than chemical inhibition of HIF-1α [89]. For example, negatively charged HIF-1α siRNA (si-HIF1α) was adsorbed on the surface of a cationic *ε*-polylysine co-polymer by electrostatic interaction, while gemcitabine was encapsulated in its hydrophilic core [90]. The resulting copolymer was then coated with a PEGylated lipid membrane to prevent degradation of the anti-HIF1α siRNA in serum. In vitro and in vivo experiments showed that HIF-1α transcription was inhibited, the levels of hypoxia-induced factors were reduced, and the anti-tumor effect of gemcitabine was enhanced. However, the complex structure of lipid–polymer hybrid NPs did not allow the control of their particle size, limiting their application only to passive targeting [91]. Therefore, gemcitabine and anti-HIF1α siRNA were co-loaded into GE11 peptide-conjugated cationic liposomes in order to actively target the epidermal growth factor receptor, which is overexpressed on the surface of cancer cells [92]. Although this approach significantly enhanced targeting ability, anti-HIF1α siRNA may be unstable on the particle surface, and the cationic liposomes themselves may be cytotoxic.

### Transport oxygen to the tumor

Oxygen transport is considered a more direct strategy for relieving hypoxia. For example, oxygen encapsulated in microvesicles/microspheres was directly delivered to tumor hypoxia sites using ultrahigh pressure [93]. However, the microvesicles are unstable, they cannot target specific tissues, and the amount of oxygen delivered is difficult to control. Thus, this approach is mostly used in combination with other strategies, especially oxygen-consuming therapies such as collaborative acoustic power and photodynamic therapy [94–96].

Oxygen production based on the special conditions of the TME has also been reported as an effective approach. For example, NPs containing CaO_2_ and coated with acid-sensitive methacrylate copolymers were prepared to control oxygen production [97]. Although the coating protected NPs from decomposition in the circulation, they were destroyed by the acidity of the hypoxic TME. This exposed the CaO_2_, which reacted with water to generate oxygen and reduce tumor hypoxia.

Excess H_2_O_2_ in tumor cells is another important source of oxygen. MnO_2_ or catalase have been used to promote H_2_O_2_ decomposition and alleviate tumor hypoxia. Small MnO_2_ particles (~ 15 nm) were modified with HA to self-assemble into larger NPs for H_2_O_2_ depletion and oxygen production at the tumor site [98]. The hollow MnO_2_ surface was also coated with a PEGylated membrane to prevent premature clearance from the circulation and to produce oxygen in cancer cells [99]. However, PEGylation was found to accelerate clearance from the blood [100], suggesting that a polymer coating may be more effective for such NPs [101]. MnO_2_ NPs coated with cancer cell membrane proved to be more effective in targeting cancer cells, and the masking membrane allowed NPs to target tumor cells via specific plasma membrane proteins and homotypic adhesion [102]. Similarly, glucose oxidase and catalase were embedded in the cancer cell membrane-camouflaged metalloporphyrin organic framework of PCN-224 to catalyze H_2_O_2_-mediated oxygen production and synergistically enhance photodynamic therapy [103].

### Create trigger release conditions in hypoxic tissues

The third hypoxia-targeting strategy involves the generation of trigger release conditions in hypoxic tissues. Hypoxia can be exploited as a triggering condition to induce drug release specifically in tumor tissues, while preventing drug delivery in normoxic tissues and therefore side effects [104, 105]. For example, 90% of gemcitabine and erlotinib encapsulated in a hypoxia-responsive diblock copolymer poly(lactic acid)-azobenzene-poly(ethylene glycol) NP was released within 50 min under anoxic conditions, in contrast to no release under normal conditions [106]. The hypoxic area is located deep in the tumor tissue, close to the tumor core, where blood vessels are sparse, the impact of matrix accumulation is strong, and the degree of hypoxia is high [107]. Therefore, the modification of hypoxia-responsive NPs has been considered as a promising strategy to improve drug accumulation at the tumor core. For example, iRGD peptide-conjugated hypoxia-responsive liposomes were prepared, as the iRGD peptide can interact with integrins and neuropilin receptors overexpressed in tumor tissues [108]. This approach increased drug release in the deep hypoxic region of the pancreatic tumor. The iRGD peptide was also used to optimize hypoxia-responsive polymers [109].

## Challenges and potential problems

TME is undeniably critical to the progression, invasion, and metastasis of pancreatic cancer, acting as a solid shield that protects the cancer cells at all-time and seriously impedes treatment progress. Nanobiotechnology is an emerging science in biomedical engineering [20], offering an opportunity to use NPs to target TME and treat cancer, which is like a sharp spear that can break the TME. Although some problems can be settled by NPs, still many challenges and potential problems we need to face.

The size of NPs is a significant factor affecting drug delivery and penetration in the stroma. Larger-sized NPs can gather around the tumor due to the prolonged circulation time, while the TME of pancreatic cancer has a denser stroma than other tumors, limiting their penetration into tumor. Oppositely, smaller-sized NPs can easily pass through it but are easily captured by mononuclear phagocyte system (MPS) [56]. Furthermore, surface charge properties of NPs are another factor that positively charged NPs are more likely to be uptake by cells than negatively charged ones, while negatively charged NPs have better stealth effect. As previously reviewed, positively charged NPs have shown potential efficacy against pancreatic cancer metastasis and have a better ability to load nucleic acid drugs [73, 110]. In addition, the EPR effect is not nearly as significant in human tumors as it is in animal models [111], and no biomarkers specific for pancreatic cancer, which makes it hard to design advanced receptors and ligands for NPs in TME. When designing NPs targeting TME, these challenges should be addressed first.

Strategies for ECM have focused on ablating its components to break down barriers that prevent the diffusion of NPs through bioactive substances such as enzymes and peptides. Nevertheless, TME is a highly coordinated system, and this would destroy the balance with unpredictable adverse effects, such as increased tumor migration and metastasis. In addition, regulation of ECM by NPs with enzyme is mostly used to promote drug delivery, which is far from plenty for treatment. Therefore, NPs-based systems need to add extra drugs, which creates a perplexing problem that how to precisely control the proportion of two cargoes in the same NPs. On the one hand, excessive enzyme may lead to some unnecessary risks and affect the loading of anti-cancer drugs. On the other hand, small amounts of the enzyme do not show enough effects, and enzyme activity is more affected by anti-cancer drugs. Meanwhile, the combination of enzymes and NPs also needs to be taken into consideration. To this end, ECM modulation strategies by NPs should be carefully formulated and evaluated. Significantly different from ECM modulation strategies, unique angiogenesis therapy based on NPs for regulating TME of pancreatic cancer has been demonstrated to be effective [79]. Without destroying the original stroma, the composition of TME was adjusted to promote the penetration of NPs. Unfortunately, the number of studies reported in this field is extremely limited, possibly because there are some undiscovered potential risks.

Currently, the combination strategy tends to regulate the immune microenvironment [112]. However, the immune microenvironment in pancreatic cancer is a complex and sophisticated system. Our understanding of its components and complex interactions is far from enough so that even if different combination strategies are used, it is still problematic to choose a tailored solution. In addition, most of the oxygen self-production systems based on NPs need to confront the same challenge, namely sustainability of oxygen production. Stable and lasting oxygen release can better inhibit the expression of various hypoxia factors and play a role in adjuvant therapy, but this also appears to have not been noticeably resolved. Notably, human tumors which have many mutations are heterogeneous compared to animal, and TME varies from patient to patient [113]. Even though animal models can provide some valuable preclinical data, it does not mean they can be well explained and applied to human patients. Therefore, in order to better design and evaluate the nano-delivery system, it is imperative to build animal models that are more similar to human pancreatic tumors.

## Conclusions and outlook

In recent decades, research on NPs for tumor cells has been approaching saturation but has not shown impressive efficacy. Regulation of TME based on nano-delivery system has brought a glimmer of hope for the treatment of pancreatic cancer. In this review, we discussed the regulation of pancreatic cancer microenvironment by nano-delivery systems, including remodeling ECM, targeting CAFs, regulating the immune microenvironment, promoting angiogenesis, and improving hypoxia condition. To sum up, although there are still some problems to be considered in the regulation of TME by NPs, such as the physicochemical properties of NPs, the heterogeneity of TME, and some potential therapeutic risks, it is still a promising research direction to improve drug delivery and tumor treatment effect. Furthermore, cancer cells and TME are a complete and complex community, and multifunctional NPs targeting both TME and cancer cells might be useful. In the end, we expect that continuing efforts to drive the development of new NPs-based therapies and strategies to target and reshape TME that will provide more effective direct or adjuvant treatments against pancreatic cancer.

## Data Availability

Not applicable.

## Abbreviations

Bcl2: B-cell lymphoma-2
BEZ 235: 2-Methyl-2-[4-(3-methyl-2-oxo-8-quinolin-3-yl-2,3-dihydro-imidazo[4,5-c]quinolin-1-yl)-phenyl]-propionitrile
CAFs: Cancer-associated fibroblasts
CaO_2_: Calcium peroxide
CAP: Cleavable amphiphilic peptide
CREKA: Cys-Arg-Glu-Lys-Ala
CRT: Calreticulin
CSF-1/CSF-1R: Colony stimulating factor-1/colony stimulating factor-1 receptor
CTLs: Cytotoxic T lymphocytes
DCs: Dendritic cells
DOX: Doxorubicin
ECM: Extracellular matrix
ECs: Endothelial cells
FAP-α: Fibroblast-activated protein-α
GE11: A dodecapeptide (YHWYGYTPQNVI)
GNPs: Gold nanoparticles
H_2_O_2_: Hydrogen peroxide
HA: Hyaluronic acid
HAase: Hyaluronidase
HER2: Human epidermalgrowth factor receptor-2
HIF-1α: Hypoxia-inducible factor-1α
HMGB-1: High-mobility group box 1
HSA: Human serum albumin
ICD: Immunogenic cell death
IDO: Indoleamine 2,3-dioxygenase
IL: Interleukin
IND: Indoximod
iRGD: Cyclo(Cys-Arg-Gly-Asp-Lys-Gly-Pro-Asp-Cys)
MMPs: Matrix metalloproteinases
MnO_2_: Manganese dioxide
MSNPs: Mesoporous silica nanoparticles
MT: Membrane type
NPs: Nanoparticles
OXA: Oxaliplatin
PCN-224: Porphyrin Zr metal–organic framework
PDAC: Pancreatic ductal adenocarcinoma
PEG: Polyethylene glycol
PEGPH20: PEGylated recombinant human hyaluronidase PH20
PI3K-γ: Phosphatidylinositol-3 kinase-γ
PLGA: Poly(lactic-*co*-glycolic acid)
ppp dsRNA: 5′-Triphosphate double-stranded RNA
PTX: Paclitaxel
rHuPH20: Recombinant human hyaluronidase PH20
SHH: Sonic hedgehog
si-HIF1α: HIF-1α siRNA
TAMs: Tumor-associated macrophages
TGF-β: Transforming growth factor-β
TME: Tumor microenvironment
TNF-α: Tumor necrosis factor-α
Tregs: Regulatory T cells
YC-1: 3-(5'-Hydroxymethyl-2'-furyl)-1-benzylindazole
